# Standardized residency programs in China: perspectives on training quality

**DOI:** 10.5116/ijme.5780.9b85

**Published:** 2016-07-13

**Authors:** Jonathan Lio, Hongmei Dong, Yanqing Ye, Brian Cooper, Shalini Reddy, Renslow Sherer

**Affiliations:** 1 Department of Medicine, University of Chicago, USA; 2 Department of Medicine, Wuhan University School of Medicine, China

**Keywords:** Standardized residency programs, perspectives, training quality, China

## Introduction

There have been momentous changes in graduate medical education in China in the past few years. While the standardization of residency training programs has been a topic of national conversation for decades, the Chinese Medical Association under the commission of the Ministry of Health released mandatory residency training standards in 2012.^1^ The training standards were divided into four sections for each specialty: training objectives, rotation length requirements, training content, and reference material. The vast majority of its pages, however, were devoted to training content, which consisted of a list of diseases and skills to master for each specialty.

At the end of 2014, 8,500 residency programs had been established in 559 hospitals enrolling 55,000 resident physicians.^2^ The Chinese government implemented a plan for the nationwide initiation of 3-year standardized residency training programs beginning 2015.^3^ The government has stated that by 2020, any physician applying for clinical work must have completed training in one of these new residency programs. These changes affect the health of a fifth of the world’s population and may interest medical educators who wish to advance international educational practices.

Before the implementation of standardized national residency training, there were many residency programs being piloted. A survey of trainees and faculty from randomly selected hospitals in China in 2006 found that trainees and faculty perceived their residency programs for the most part met basic global standards for postgraduate medical education.^4^ However, some of the leading programs reported inadequate supervision,^5^ unstandardized teaching,^6^ and wide variations in the use of case discussions.^7^ A Chinese literature review concluded that the existing training processes suffered from a lack of standardization across different training hospitals.^8^

Despite the initiation of national standardization of residency programs and curricula in the most populous country in the world, there has been no published literature in English describing these programs. Therefore we sought to understand residents’ perceptions of their training programs at a teaching hospital in a provincial capital in China, regarding program organization, quality of clinical teaching, and teaching of competencies. We report here the lessons we learned from our surveys of the residents.

### Program organization

Residents noted important limitations to the organization of their training programs.  While regular case discussions were present, they most often occurred only once a week or less, which is likely too infrequent to provide an environment conducive to learning, especially since rounds are often teacher-centered and involve passive learning for the residents.^9^ Even less frequent were journal clubs, which can be an invaluable resource to counteract uncritical attitudes toward medical tradition by encouraging trainees to appraise evidence-based recommendations for changes to current standards of practice.^10^ Furthermore, not having access to previous performance evaluations, as reported by more nearly half of the residents, denied resident trainees the opportunity to critically evaluate their approach to learning.

### Clinical teaching quality

There were important areas for improvement in the quality of clinical teaching as well. Residents were unsatisfied with the amount of faculty supervision, amount of teaching, amount of time devoted to organized learning, and degree of independence in patient care. Only half of them said they were encouraged to think during rounds. They suggested that faculty lacked teaching skills that promote stimulating clinical conversations with residents, encourage the habitual use of current medical literature in clinical decision-making, or support regular constructive feedback throughout the training process. Residents also thought they had inadequate supervision from attendings but also not enough autonomy. It may be beneficial to find ways to support teaching attendings by providing training in teaching methods and by protecting their time dedicated to residents.

### Teaching of competencies

Residents were unable to differentiate well between different competency categories, which would not be surprising since the Chinese graduate medical education system currently lacks a mature competency framework to guide the education of residents.^11^ While the ACGME has defined six core competencies and many associated sub-competencies in an attempt to cover the full scope of a physician’s practice of medicine, the Chinese national curricular document^1^ focuses on listing the diseases and disease-specific skills that trainees should master according to each specialty. It should be a national priority to develop consensus on the combination of knowledge, skills, and attitudes required of a physician in China and the developmental milestones on the pathway to proficiency.^12^^,^^13^

## Conclusions

China’s recent efforts to standardize residency training reflect a tremendous desire to invest in the health of its people. The national government has taken a large step forward in issuing training standards for residency programs, but these standards have not necessarily created quality programs. While residents at this particular teaching hospital perceived the essential elements of a program in place, they also described many areas for additional development. Furthermore, given the recent creation and standardization of programs across the country, we suspect that many other training programs may be facing the same problems. As this paper and previously published Chinese literature suggest, residency training still has a long way to go before it is truly “standardized”.

### Conflict of Interest

The authors declare that they have no conflict of interest.
